# Femtosecond X-ray solution scattering reveals that bond formation mechanism of a gold trimer complex is independent of excitation wavelength

**DOI:** 10.1063/1.4948516

**Published:** 2016-04-29

**Authors:** Kyung Hwan Kim, Jong Goo Kim, Key Young Oang, Tae Wu Kim, Hosung Ki, Junbeom Jo, Jeongho Kim, Tokushi Sato, Shunsuke Nozawa, Shin-ichi Adachi, Hyotcherl Ihee

**Affiliations:** 1 Center for Nanomaterials and Chemical Reactions, Institute for Basic Science (IBS), Daejeon 305-701, South Korea; 2 Department of Chemistry, KAIST, Daejeon 305-701, South Korea; 3 Department of Chemistry, Inha University, Incheon 402-751, South Korea; 4 Institute of Materials Structure Science, High Energy Accelerator Research Organization (KEK), 1-1 Oho, Tsukuba, Ibaraki 305-0801, Japan

## Abstract

The [Au(CN)_2_^−^]_3_ trimer in water experiences a strong van der Waals interaction between the d^10^ gold atoms due to large relativistic effect and can serve as an excellent model system to study the bond formation process in real time. The trimer in the ground state (S_0_) exists as a bent structure without the covalent bond between the gold atoms, and upon the laser excitation, one electron in the antibonding orbital goes to the bonding orbital, thereby inducing the formation of a covalent bond between gold atoms. This process has been studied by various time-resolved techniques, and most of the interpretation on the structure and dynamics converge except that the structure of the first intermediate (S_1_) has been debated due to different interpretations between femtosecond optical spectroscopy and femtosecond X-ray solution scattering. Recently, the excitation wavelength of 267 nm employed in our previous scattering experiment was suggested as the culprit for misinterpretation. Here, we revisited this issue by performing femtosecond X-ray solution scattering with 310 nm excitation and compared the results with our previous study employing 267 nm excitation. The data show that a linear S_1_ structure is formed within 500 fs regardless of excitation wavelength and the structural dynamics observed at both excitation wavelengths are identical to each other within experimental errors.

## I. INTRODUCTION

Metal complexes with central metal atoms of gold or silver have attracted much interest due to their unique property called aurophilicity.^1–7^ The aurophilicity, which is a relativistic effect, causes strong van der Waals interactions between gold atoms with the d^10^ electron configuration. As a result, interatomic distance between a pair of gold atoms becomes significantly smaller than the sum of van der Waals radii (∼3.8 Å) of two gold atoms. Among various compounds that exhibit the aurophilic interactions, a gold oligomer complex, [Au(CN)_2_^−^]_n_, has been studied intensely.^8–12^ In a highly concentrated solution of Au(CN)_2_^−^ monomers, due to the aurophilic interactions, Au atoms are weakly bound to each other even without covalent bonds and a dimer or trimer aggregate complex of Au(CN)_2_^−^ is formed. On photoexcitation of the complex, one electron in an antibonding orbital is excited to a bonding orbital, leading to the formation of covalent bonds among gold atoms. Therefore, [Au(CN)_2_^−^]_n_ can serve as an excellent model system for studying the dynamics of bond formation in solution because (i) the reaction partners are prepared in close proximity within the same solvent cage and (ii) the reaction can be synchronized with the laser excitation.

**FIG. 1.**
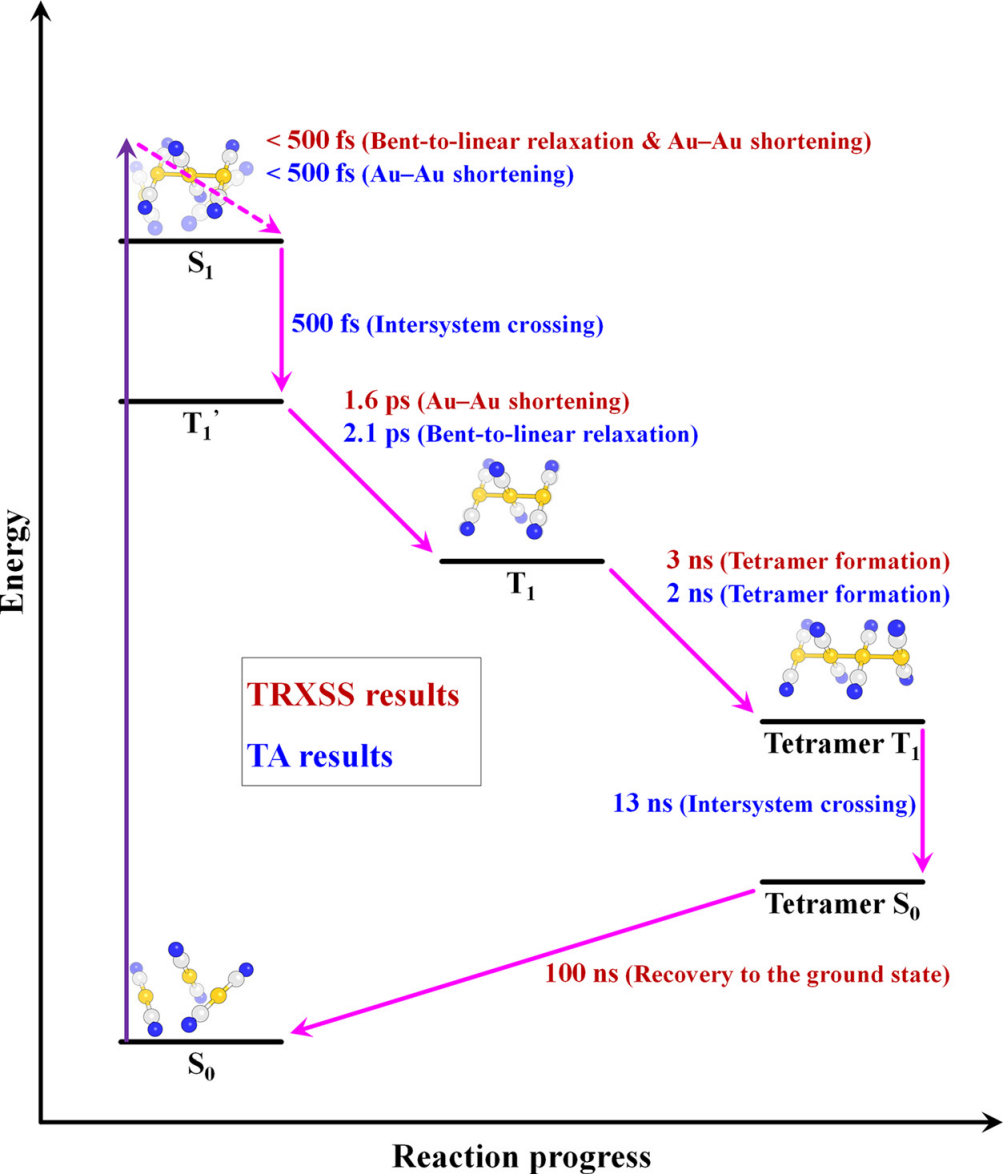
Reaction pathways of photoinduced bond formation in [Au(CN)_2_^−^]_3_. Observations from TRXSS (red) and TA (blue) experiments are compared. Reaction mechanisms revealed by the two experiments are in good agreement with each other, except for the structural assignments of early kinetics. The 500 fs component was assigned to the S_1_-toT_1_ intersystem crossing in the TA study but was not observed by TRXSS. This discrepancy suggests that the intersystem crossing does not involve any significant structural change detectable by TRXSS, and thus we termed the initially formed T_1_ state as T_1_′, which is structurally indistinguishable from S_1_.

Recently, the dynamics of Au–Au bond formation in a Au trimer complex, [Au(CN)_2_^−^]_3_, in solution was investigated using transient absorption (TA) spectroscopy.^10^ Temporal changes of TA spectra induced by photoinduced formation of Au–Au covalent bonds were observed and described by kinetic components with time constants of 500 fs, 2 ps, and 2 ns. In that TA study, the kinetic component of 2 ps time constant was assigned to conformational change from bent to linear structure, but that assignment was disputed by an *ab initio* molecular dynamics simulation, which claimed that the bent-to-linear structural transition occurs within 500 fs.^11^ Later, time-resolved X-ray solution scattering (TRXSS) was performed on [Au(CN)_2_^−^]_3_ at an X-ray free electron laser (XFEL) and confirmed that the bent-to-linear transition is completed within 500 fs.^12,13^ TRXSS, which is also called time-resolved X-ray liquidography (TRXL), is sensitive to global molecular structure and thus can directly characterize the structure of transient species with sub-Å spatial resolution.^14–21^ In contrast, TA signal is not directly related to the molecular structure and thus cannot determine the structure of transient species unambiguously. The reaction pathways of photoinduced bond formation in [Au(CN)_2_^−^]_3_ are summarized in Figure 1 with the assignments of kinetics from the TA and TRXSS studies. We note that the 500 fs component was assigned to the S_1_-to-T_1_ intersystem crossing in the TA study but was not observed by TRXSS. This discrepancy suggests that the intersystem crossing, if the assignment in the TA study is correct, does not involve any significant structural change detectable by TRXSS, and thus we termed the initially formed T_1_ state as $T_{1}^{\prime }$, which is structurally indistinguishable from S_1_.

**FIG. 2.**
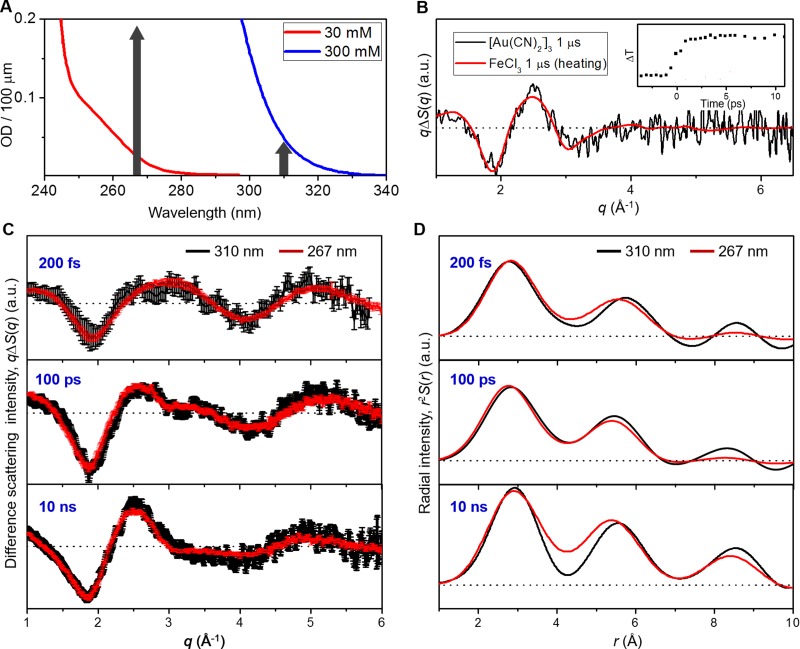
(a) Absorption spectra of Au(CN)_2_^−^ dissolved in water solution at two different concentrations, 30 mM (red) and 300 mM (blue). Two different excitation wavelengths (310 nm and 267 nm) are indicated by vertical arrows. (b) Comparison of the difference scattering curve of the [Au(CN)_2_^−^]_3_ solution at 1 *μ*s time delay (black) and the difference scattering curve for solvent heating (red) obtained from a separate heating experiment on FeCl_3_ solution. Since the two difference scattering curves have the same shape, the total amount of heat dissipated in the sample solution was determined by scaling these two curves. Temporal change in temperature determined from the FeCl_3_ experiment is shown in the inset and was used for subtraction of heating contribution from the difference scattering curves of [Au(CN)_2_^−^]_3_ solution. (c) Comparison of the time-resolved difference scattering curves measured with excitation at two different wavelengths, 310 nm and 267 nm. For simplicity, the scattering curves at three representative time delays, 200 fs (top panel), 100 ps (mid panel), and 10 ns (bottom panel), are shown. Difference scattering curves measured with the excitation at 310 nm were multiplied by two for comparison with the ones measured with the excitation at 267 nm. It can be clearly seen that the difference scattering curves measured with excitation at 310 nm (black) and 267 nm (red) have the same shape within the experimental error, indicating the same kinetics and intermediate structures with 310 nm and 267 nm excitation. (D) Comparison of the radial distribution functions, *r*^2^*S*(*r*), measured with excitation at two different wavelengths, 310 nm and 267 nm.

However, in a subsequent TA study on [Au(CN)_2_^−^]_2_ dimers that are dominantly formed in a solution of low concentration (30 mM),^22^ it was claimed that the discrepancy on the dynamics of bent-to-linear transition of [Au(CN)_2_^−^]_3_ trimers between the TA and TRXSS studies can be attributed to the difference in excitation wavelength used in the TA (310 nm) and TRXSS (267 nm) measurements. Figure 2(a) shows the absorption spectra of Au(CN)_2_^−^ solutions in water at two different concentrations (30 mM and 300 mM). As the initial concentration of Au(CN)_2_^−^ monomers increases, the absorption spectrum shifts towards longer wavelengths, indicating the shift of the equilibrium between dimer (dominant at 30 mM) and trimer (dominant at 300 mM) species of Au(CN)_2_^−^. Excitation of the 300 mM solution at 310 nm, which corresponds to the tail of the trimer absorption, can selectively initiate the photoinduced Au–Au bond formation of the trimer species. In the previous TA study,^10^ the 300 mM solution was excited at the wavelength of 310 nm. In contrast, excitation of the 300 mM solution at 267 nm, which corresponds to the main peak of the trimer excitation, can initiate the photoinduced Au–Au bond formation of the trimer species with a high yield but also excite the dimers (only with a low yield, though), which may complicate the analysis of the transient signal for the Au–Au bond formation of [Au(CN)_2_^−^]_3_ trimer. In the previous TRXSS study,^12^ the 300 mM solution was excited at the wavelength of 267 nm to achieve a higher yield of photoinduced Au–Au bond formation and thus a higher signal-to-noise ratio. However, in the analysis of the TRXSS data, the TRXSS data were consistent with the assumption that the dimer contribution to the TRXSS signal is negligible based on the theoretical estimation of the dimer contribution to the time-resolved scattering signal using the equilibrium constants and the extinction coefficients of the trimer and dimer species. In the later TA study,^22^ the TA experiment was performed on the 30 mM solution, where the dimers are dominant and the trimers are nearly absent, with photoexcitation at 267 nm so that the Au–Au bond formation dynamics of the dimers can be selectively probed. From that study, it was found that the kinetic component of 2 ps time constant is absent in the bond formation dynamics of the dimer species. Based on this observation, the authors claimed that the 2-ps kinetic component observed only in the TA signals of trimers corresponds to the bent-to-linear transition of [Au(CN)_2_^−^]_3_ trimer and hinted that the previous TRXSS signal could be contaminated by the contribution of dimer species.

To resolve this controversy, in this work, we investigate the reaction mechanism of photoinduced Au–Au bond formation of [Au(CN)_2_^−^]_3_ trimer by performing femtosecond TRXSS measurement on the 300 mM solution of Au(CN)_2_^−^ with 310 nm laser excitation. Under this experimental condition, the possibility of dimer excitation becomes substantially smaller than with 267 nm excitation and [Au(CN)_2_^−^]_3_ trimers can be excited selectively. Then, we can examine whether the reaction mechanism of the Au–Au bond formation changes with excitation wavelength by comparing the reaction mechanisms obtained with excitation at 310 nm and 267 nm. Based on this result, we can validate the assumption that the contribution of the dimer excitation to the TRXSS signal of [Au(CN)_2_^−^]_3_ is negligible, which was used for analyzing the TRXSS data measured with 267 nm laser excitation in our previous TRXSS study.^12^

## II. EXPERIMENTAL

The TRXSS measurement and data analysis were performed by using the experimental and analysis protocols described in Secs. II A–II F and our previous publications.^12,16,23,24^ The TRXSS data were collected at the BL3 beamline of SACLA and the NW14A beamline of KEK. X-ray pulses with sub-100 fs duration generated from SACLA were used for measuring the data at early time delays (from −800 fs to 100 ps) and X-ray pulses with 100 ps duration generated from KEK were used for measuring the data at late time delays (from 100 ps to 1 *μ*s).

### A. TRXSS data collection at SACLA

The TRXSS measurement at early time delays (from −800 fs to 100 ps) was performed at the BL3 beamline of SACLA. The laser pulses with the pulse duration of ∼100 fs and the center wavelength of 310 nm were focused onto a spot of 0.095 × 0.12 mm^2^ size, giving the fluence of ∼2 mJ/mm^2^. The X-ray pulses generated from an XFEL at SACLA had the pulse duration of <100 fs, the center energy of 15 keV, and the photon flux of ∼10^11^ photons per pulse.^25–27^ The laser pulses were used to initiate the Au–Au bond formation, and the X-ray pulses were used to monitor photoinduced structural changes. The scattering patterns were measured with an area detector (Rayonix LX255-HS). A solution of gold oligomer complex, [Au(CN)_2_^−^]_n_, dissolved in water at the concentration of 300 mM was circulated through a sapphire nozzle with 100 *μ*m-thick aperture. To obtain time-resolved scattering signal, the difference scattering curves, Δ*S*(*q*), were obtained by subtracting the scattering curve measured at −5 ps from the scattering curves measured at positive time delays. Each scattering image was acquired by accumulating ∼80 X-ray shots and, at each time delay, ∼240 images were acquired at each time delay to achieve a high signal-to-noise ratio enough for data analysis. The scattering curves were measured at the following time delays: −5 ps, −800 fs, −300 fs, 200 fs, 1.2 ps, 1.7 ps, 3.2 ps, 5.2 ps, 10 ps, 20 ps, 30 ps, 50 ps, and 100 ps. The time zero was determined by fitting the time trace of the difference scattering data for the sample solution with a convolution of a Gaussian function representing an instrument response function (IRF) and a sum of exponential functions representing the transition dynamics among intermediate states. The overall jitter between the laser and X-ray pulses was estimated to be less than 1.4 ps, which corresponds to a full-width half maximum (FWHM) value of the fitted IRF and the time resolution of our measurement. The determined time resolution is longer than that of our previous work at 267 nm, because the weaker difference scattering signal with 310 nm excitation required much longer data collection.

### B. TRXSS data collection at KEK

TRXSS measurement at late time delays (100 ps–1 *μ*s) was performed at the NW14A beamline of KEK.^28^ The laser pulses with the center wavelength of 310 nm were focused onto a spot of 0.343 × 0.492 mm^2^ size, giving the fluence of ∼0.2 mJ/mm^2^. The X-ray pulses generated from a synchrotron at KEK had the center energy of 15.6 keV, the photon flux of 2 × 10^8^ photons per pulse, and the pulse duration of ∼100 ps. The laser pulses were used to initiate the Au–Au bond formation, and the X-ray pulses were used to monitor photoinduced structural changes. The scattering patterns were measured with an area detector (MarCCD165, Mar USA). A solution of gold oligomer complex, [Au(CN)_2_^−^]_n_, dissolved in water at the concentration of 300 mM was circulated through a sapphire nozzle with 300 *μ*m-thick aperture. To obtain time-resolved scattering signal, the difference scattering curves, Δ*S*(*q*), were obtained by subtracting the scattering curve measured at −5 ns from the scattering curves measured at positive time delays. Each scattering image was acquired by accumulating ∼4000 X-ray shots and, at each time delay, ∼80 images were acquired to achieve a high signal-to-noise ratio enough for data analysis. The scattering curves were measured at the following time delays: −3 ns, −150 ps, 100 ps, 150 ps, 300 ps, 1 ns, 3 ns, 10 ns, 30 ns, 100 ns, 300 ns, and 1 *μ*s.

### C. Removal of the solvent contribution

In order to obtain scattering signals only from the Au–Au bond formation, the scattering arising from solvent heating was subtracted from the experimental scattering data. The water heating signal was obtained by a separate solvent heating experiment on FeCl_3_ solution at the same time delays used for the [Au(CN)_2_^−^]_3_ solution. Since the difference scattering curve of the [Au(CN)_2_^−^]_3_ solution at 1 *μ*s time delay has the same shape as the one for solvent heating, as shown in Figure 2(b), due to the reversibility of the reaction, the amount of heat dissipated in the sample solution was determined by scaling these two curves. The resultant difference scattering curve obtained by subtracting the solvent heating contribution can be regarded as the solute-only term because the contribution of the cage term (=solute-solvent cross term) is negligibly small.^12^

### D. Sine-Fourier transformation of *qΔS*(*q*)

The difference scattering intensities in *q*-space, *qΔS*(*q*), can be converted to difference radial distribution functions (RDFs) in real space, *r*^2^Δ*S*(*r*), by sine-Fourier transformation

$$r^{2}\Delta S(r,t)=\frac{r}{2\pi^{2}}\int_{0}^{\infty }q\Delta S(q,t)\text{sin}(qr)e^{-q^{2}\alpha }dq,$$ (1)

where the constant α (α = 0.03 Å^2^) is a damping term that accounts for the finite *q* range in the experiment. Difference RDFs represent the change of Au–Au interatomic distances in the molecules participating in the reaction and thus provide an intuitive picture of change in molecular structure.

### E. Structural and kinetic analyses

Theoretical RDFs were calculated by the sum of multiple RDFs which corresponds to the Au–Au pairs

$$r^{2}S_{theory}(r)=\sum_{i=1}^{n}\frac{r}{2\pi^{2}}\int_{0}^{\infty }qF_{Au}^{2}(q)\frac{\;\text{sin}\;qR_{i}}{qR_{i}}\text{sin}(qr)e^{-q^{2}\alpha }dq,$$ (2)

where *F_Au_* is the atomic form factor of the gold atom and *R*_i_ is the Au–Au distance for the *i*th pair of Au atoms. For the dimer, trimer and the tetramer, *n* was set to be 1, 3, and 6, respectively.

In our previous TRXSS study employing 267 nm laser excitation,^12^ we used Au–Au distances (*R*_i_) and three time constants as free fitting parameters for structural and kinetic analyses, respectively. In this work, we just used the same intermediate structures and kinetic parameters determined in our previous TRXSS study instead of re-optimizing them (see Section III A for details).

### F. Determination of dimer contribution

In order to determine the relative contribution of dimers to the TRXSS signal measured from the 300 mM solution of Au(CN)_2_^−^, we fitted an experimental RDF (or scattering curve) at an early time delay with a theoretical RDF (or scattering curve) that considers the contributions of trimers and dimers as follows:

$$\begin{array}{c} r^{2}S_{theory}(r)=f\sum_{i=1}^{n}\frac{r}{2\pi^{2}}\int_{0}^{\infty }qF_{Au}^{2}(q)\frac{\;\text{sin}\;qR_{i}}{qR_{i}}\text{sin}(qr)e^{-q^{2}\alpha }dq\\ +(1-f)\frac{r}{2\pi^{2}}\int_{0}^{\infty }qF_{Au}^{2}(q)\frac{\;\text{sin}\;qR_{\text{dimer}}}{qR_{\text{dimer}}}\text{sin}(qr)e^{-q^{2}\alpha }dq. \end{array}$$ (3)

In the above equation, two additional fitting parameters were considered compared with Eq. (2). The parameter *f* is the relative fraction of the trimer excitation which varies from 0 to 1 and *R*_dimer_ is the bond length of the excited dimer species. The maximum likelihood estimation (MLE) with the χ^2^ estimator was employed^29^ with six variable parameters. The chi-square (χ^2^) is given by the following equation:

$$\chi^{2}(R_{1},R_{2},R_{3},f,\;R_{\text{dimer}},A)=\frac{1}{N-p-1}\sum_{i}\frac{{\left(S_{theory}(r_{i})-S_{\;\text{exp}\;}(r_{i})\right)}^{2}}{\sigma_{i}^{2}},$$ (4)

where *A* is the scaling factor between the number of excited molecules and the signal intensity, *N* is the total number of *r* points (= 500), *p* is the number of fitting parameters (= 6), and *σ_i_* is the standard deviation. The calculation was performed by the MINUIT^30^ library, and the error values were provided by the MINOS algorithm in MINUIT.

## III. RESULTS AND DISCUSSION

**FIG. 3.**
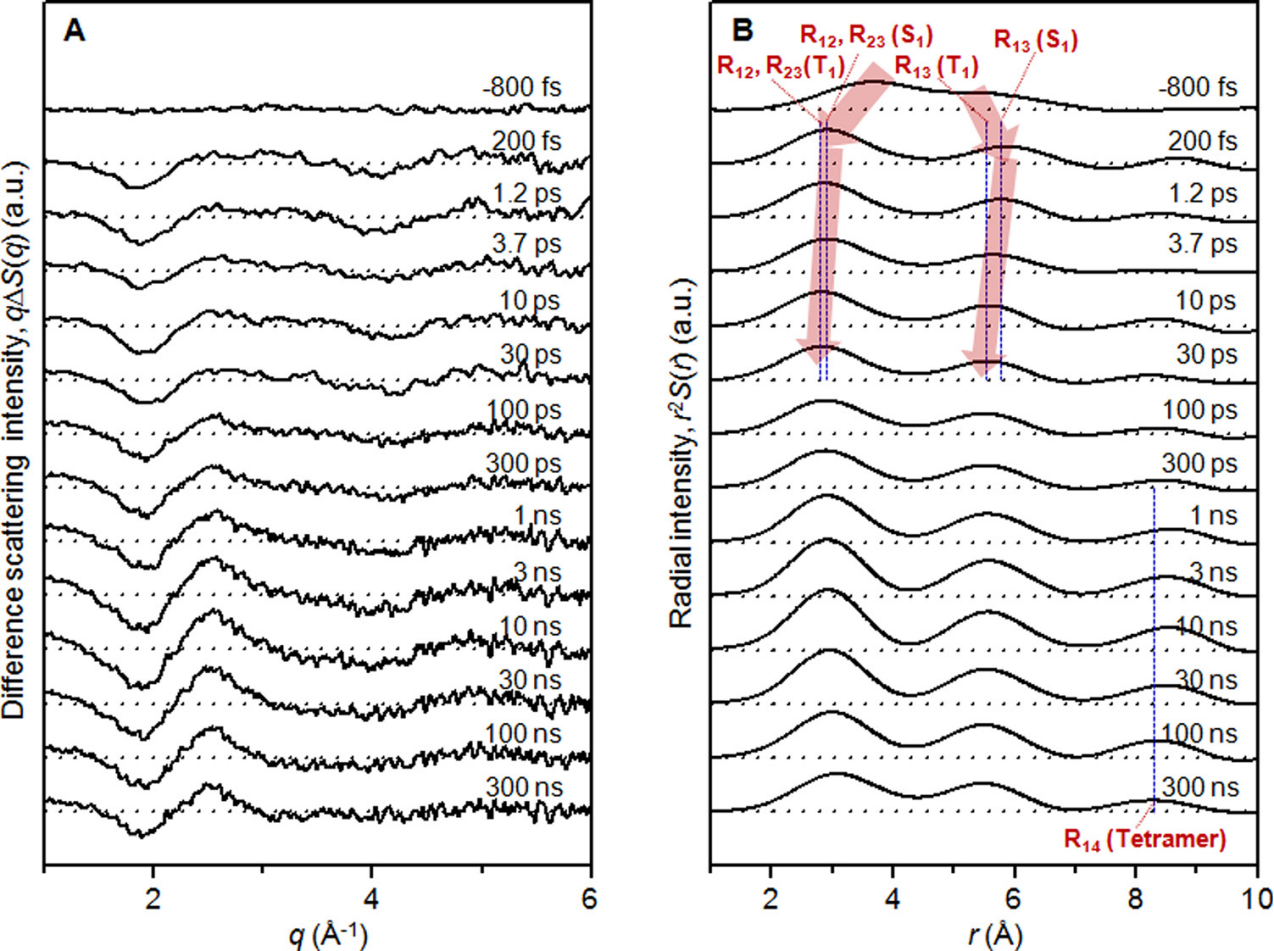
(a) Experimental difference scattering curves, *qΔS*(*q*), at various time delays from −800 fs to 300 ns. The curves clearly exhibit oscillatory features that vary with time. (b) Radial distribution functions (RDFs), *r*^2^*S*(*r*), obtained by sine-Fourier transformation of *qΔS*(*q*) in (A) and addition of the RDF of the S_0_ state. The blue dashed lines indicate the locations of Au–Au distances of reaction intermediates determined in the previous TRXSS study employing 267 nm laser excitation.^12^

In Figures 2(c) and 2(d), difference scattering curves and corresponding radial distribution functions obtained from the 300 mM solution of Au(CN)_2_^−^ with 310 nm (black) and 267 nm (red) excitation are compared at three representative time delays (200 fs, 100 ps, and 10 ns). The scattering patterns at each time delay are very similar to each other within the experimental error, indicating the same kinetics and intermediate structures with excitation at 310 nm and 267 nm. Although the difference scattering curves at three representative time delays are shown in Figure 2(c) for simplicity, the comparison at other time delays gives the same conclusion. Experimental difference scattering curves, *qΔS*(*q*,*t*), in the entire time range are shown in Figure 3(a). The data were analyzed by the protocol described in the Experimental section and the results are shown in Secs. III A–III D.

### A. Structural and kinetic parameters of chemical species involved in the reaction

Structural and kinetic parameters of reaction intermediates (S_0_, S_1_, T_1_, and tetramer) involved in the photoinduced bond formation of [Au(CN)_2_^−^]_3_ trimer initiated by laser excitation at 267 nm were determined in our previous TRXSS study^12^ as summarized in Tables I and II, respectively. The details of the structural and kinetic analysis are described in our previous work. Based on the close similarity between the difference scattering curves measured with excitation at two different wavelengths (310 nm and 267 nm) as shown in Figure 2(c), we can expect the same kinetics and intermediate structures for bond formation initiated by 310 and 267 nm excitation. Therefore, in this work, we used the same intermediate structures and kinetic parameters determined in the previous TRXSS study^12^ instead of re-optimizing them.

### B. Population kinetics and associated changes in Au–Au interatomic distance

The difference scattering curves in Figure 3(a) exhibit oscillatory features that vary with time. Those oscillatory patterns and their temporal changes reflect the structures of reaction intermediates and the temporal evolution of Au–Au interatomic distances. In order to extract only the dynamics of Au–Au interatomic distances, the contribution of solvent heating was subtracted from the experimental scattering curves using the protocols described in Section II C.

An intuitive picture of structural changes involved in the bond formation process can be obtained by converting the difference scattering intensities in *q*-space, *qΔS*(*q*), into difference radial distribution functions (RDFs) in real space, *rΔS*(*r*), using sine-Fourier transformation. Since *qΔS*(*q*) represent the difference between the scattering intensities of newly formed intermediate species and the depleted ground state, the excitation fraction (or the fraction of ground-state depletion) can be obtained from *qΔS*(*q*). To emphasize the scattering features of transient intermediate species associated with bond formation, we added the RDF of the ground (S_0_) state weighted by the excitation fraction to the difference RDFs at all time delays and obtained RDFs, *r*^2^*S*(*r*,*t*), as shown in Figure 3(b). These RDFs represent the temporal evolution of Au–Au interatomic distances in the molecules participating in the reaction. By analyzing the RDFs, we can directly identify the chemical species involved in the reaction and quantify the population change of each species during the progress of the reaction.

Two peaks at ∼3.0 and ∼5.5 Å are distinct at early time delays up to 300 ps, and another peak at ∼8.0 Å appears at time delays of 1–10 ns. To facilitate the comparison between the bond formation processes initiated by excitation at 310 nm and 267 nm, we used blue dashed lines in Figure 3(b) to indicate the locations of Au–Au distances for all the intermediate species (R_12_ and R_13_ of S_1_ state, R_12_ and R_13_ of T_1_ state, and R_14_ of tetramer, where R_ij_ stands for the internuclear distance between the ith and jth Au atoms.) determined in our previous study employing 267 nm laser excitation.^12^ Although the RDFs have small artifactual oscillation at *r* > 8 Å due to relatively poor signal-to-noise ratio of the TRXSS data obtained with 310 nm excitation, temporal change of Au–Au interatomic distances manifested in the RDFs obtained with 310 nm excitation can be well explained by the Au–Au distances of the intermediates determined in our previous study employing 267 nm excitation. Within our time resolution (∼200 fs), the initial ground (S_0_) state, which has loosely bound Au atoms and a bent structure, impulsively changes to the S_1_ state, which has much shorter Au–Au distances and a linear structure. From 200 fs to 10 ps, R_12_ and R_13_ decrease further, indicating the formation of the T_1_ state. After 100 ps, the peak at ∼8.0 Å, which corresponds to the R_14_ of the tetramer, grows up until 10 ns. After 10 ns, the RDF gradually returns to the RDF of the S_0_ state.

### C. Species-associated RDFs and population change of each species

**FIG. 4.**
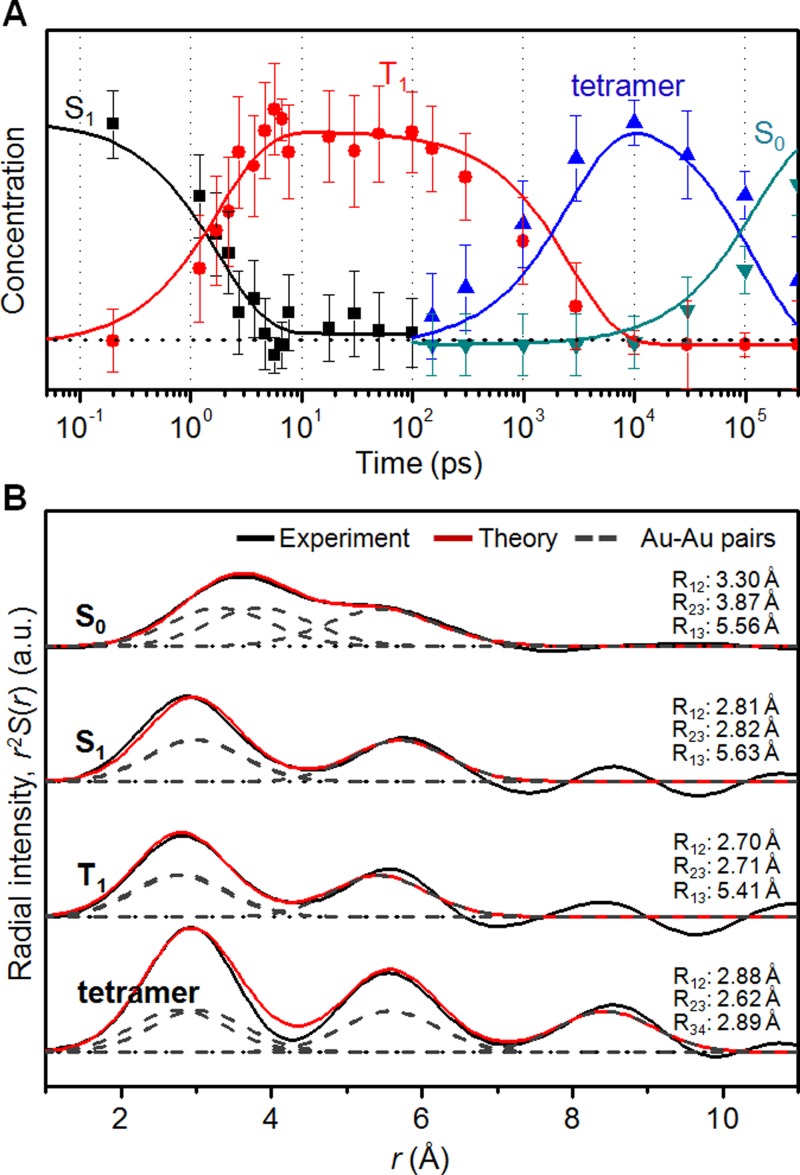
(a) Time-dependent population changes (points with error bars) of the four reaction intermediates after photoexcitation of the [Au(CN)_2_^−^]_3_ trimer at 310 nm. The name of each intermediate species is indicated above each trace. The same kinetic parameters determined in the previous TRXSS study employing 267 nm laser excitation^12^ were used to construct the exponential fits (lines) of the time-dependent population changes. The symbols represent the concentrations of intermediate species determined by fitting the experimental data at each time delay by a linear combination of species-associated radial distribution functions. The error bar at each time delay represents standard error of the fit. (b) Species-associated radial distribution function, *r*^2^*S*(*r*), of the four intermediates (black) obtained by singular value decomposition and principal component analysis. Theoretical RDFs (red) built with the structures determined in the previous TRXSS study employing 267 nm laser excitation^12^ are shown together. The Au–Au distances of each intermediate used for generating the theoretical RDFs are shown on right.

Species-associated RDFs for the four reaction intermediates (S_0_, S_1_, T_1_, and tetramer) and their time-dependent population changes were obtained by singular value decomposition (SVD) and principal component analysis (PCA) as was done in our previous publication.^12^ Time-dependent population changes of the four transient intermediates after photoexcitation of the [Au(CN)_2_^−^]_3_ trimer at 310 nm and their exponential fits are shown in Figure 4(a). The same kinetic parameters (1.6 ps, 3 ns, and 100 ns listed in Table II) determined in the previous study employing 267 nm laser excitation^12^ were used to construct the exponential fits of the time-dependent population changes. Although the time-dependent population changes show much larger errors compared with the data obtained with 267 nm excitation due to relatively low signal-to-noise ratio of the data obtained with 310 nm excitation, the population changes are well described by the same kinetic parameters determined from the ones obtained with 267 nm excitation.

Species-associated RDFs, *r*^2^*S*(*r*), of the four transient species determined from the experimental data obtained with 310 nm excitation are shown in Figure 4(b) together with theoretical RDFs calculated from the structures determined in the previous TRXSS study employing 267 nm excitation (see Table I). Although the RDFs have small artifactual oscillation at *r* > 8 Å due to relatively poor signal-to-noise ratio of the TRXSS data obtained with 310 nm excitation, the experimental and theoretical RDFs are in good agreement. These results suggest that the TRXSS data measured with excitation at the two different wavelengths are well described by the same kinetics and intermediate structures. Therefore, we conclude that the reaction mechanism of photoinduced Au–Au bond formation of [Au(CN)_2_^−^]_3_ trimer is independent of excitation wavelength.

### D. Determination of dimer contribution

**FIG. 5.**
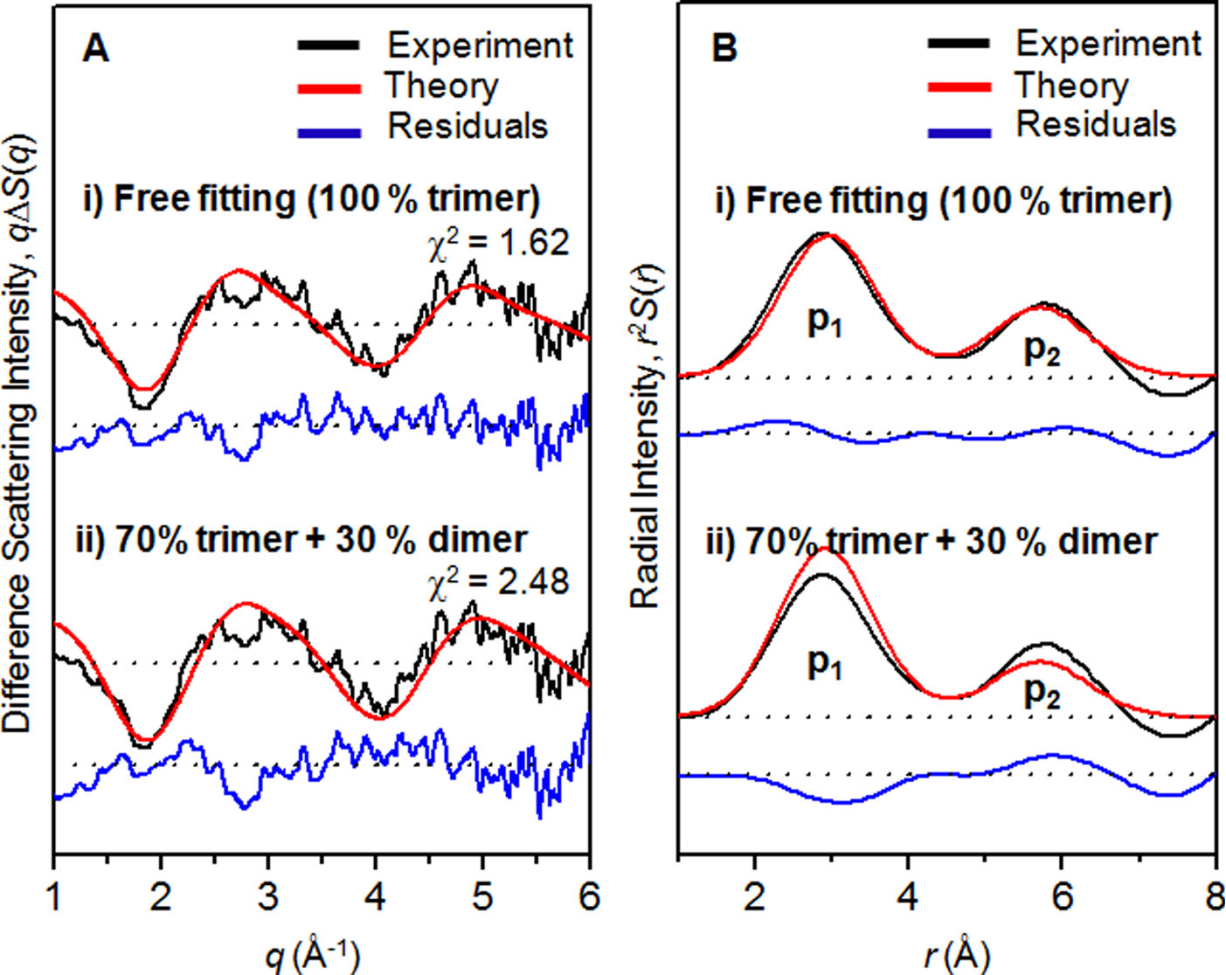
(a) Experimental difference scattering curve at 200 fs time delay (black) obtained with 310 nm excitation and its fit by a theoretical scattering curve (red) that considers the contributions of trimers and dimers. The residual (blue) obtained by subtracting the theoretical scattering curve from the experimental scattering curve is shown at the bottom. The best fit was obtained when the contribution of dimer excitation converges to zero. When we force the relative fraction of dimer excitation to be 30%, the fitting quality deteriorates significantly. (b) Experimental (black) and theoretical (red) RDFs obtained by sine-Fourier transformation of the scattering patterns shown in (a).

In Secs. III A–III C, we showed that the reaction mechanism of photoinduced Au–Au bond formation of [Au(CN)_2_^−^]_3_ trimer is not affected by excitation wavelength. This finding indicates that the contribution of [Au(CN)_2_^−^]_2_ dimers to the TRXSS signal measured from the 300 mM solution of Au(CN)_2_^−^ must be negligible, regardless of excitation wavelength, as was assumed in our previous TRXSS study employing 267 nm excitation. To confirm this finding, we estimated the relative contribution of dimers to the TRXSS signal measured from the 300 mM solution of Au(CN)_2_^−^. To do so, considering that the lifetime of the excited dimer is as short as 26 ps,^22^ we fitted the experimental difference scattering curve at the earliest time point (200 fs) with a theoretical scattering curve that considers the contributions of trimer and dimer species as described in Section II F. The reduced chi-square value that represents the deviation between the experimental and theoretical scattering curves was minimized by optimizing the relative fraction of the trimer and dimer excitation as a free fitting parameter. The result of the fitting analysis is shown in Figure 5. From the fitting, the best fit was obtained when the contribution of the dimer excitation converges to zero (χ^2 ^= 1.62), indicating that the TRXSS signal measured with 310 nm laser excitation is dominated by the contribution of the trimer excitation with negligible contribution from dimers. If we force the relative fraction of the dimer excitation to be a nonzero value, the fitting quality deteriorates increasingly with the increase of dimer contribution. For example, when the fraction of dimer excitation is 30%, the chi-square (the figure of merit) value is 2.48. Even when the structural parameters of dimer and trimer are used as fitting parameters that are freely variable together with the contributions of the dimer and trimer, the contribution of the dimer still converges to zero for the best fits.

The contribution of dimers can be estimated more intuitively in the RDF in *r*-space as shown in Figure 5(b). As discussed in Section III B, two peaks (p_1_ and p_2_) are distinct in the experimental RDFs. Based on the peak positions (Au–Au distances), p_1_ can be assigned to either R_12_ or R_23_ of trimer species or the Au–Au bond length of dimer species while p_2_ must be the signature of R_13_ of trimer species. Then, the ratio between the intensities of p_1_ and p_2_ (p_1_/p_2_) can serve as a direct measure of the dimer/trimer excitation ratio. If there is any contribution from dimers, the intensity of p_1_ increases and the p_1_/p_2_ ratio becomes larger than 2, which is expected for trimers. Because the experimental RDF shows the p_1_/p_2_ ratio close to 2, the contribution of the dimer excitation must be negligible. This result of fitting analysis is in good agreement with the negligible extinction coefficient of dimer species at 310 nm.

**FIG. 6.**
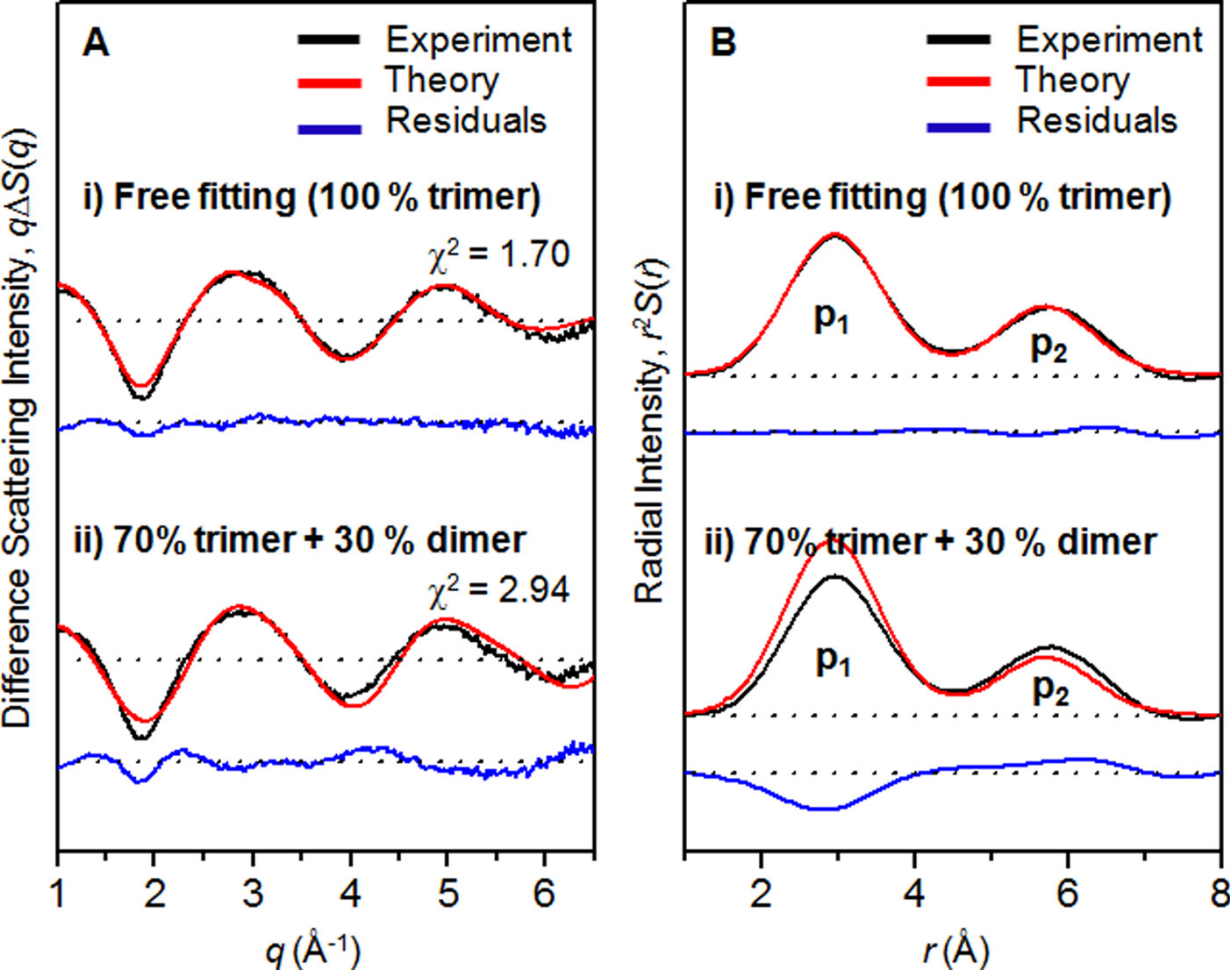
(a) Experimental difference scattering curve at 200 fs time delay (black) obtained with 267 nm excitation and its fit by a theoretical scattering curve (red) that considers the contributions of trimers and dimers. The residual (blue) obtained by subtracting the theoretical scattering curve from the experimental scattering curve is shown at the bottom. The best fit was obtained when the contributions of dimer excitation converges to zero. When we force the relative fraction of dimer excitation to be 30%, the fitting quality deteriorates significantly. (b) Experimental (black) and theoretical (red) RDFs obtained by sine-Fourier transformation of the scattering patterns shown in (a).

We also applied the same fitting analysis to the experimental difference scattering curves measured with 267 nm laser excitation presented in our previous TRXSS study.^12^ The result of the fitting analysis is shown in Figure 6. As was the case for the TRXSS data measured with 310 nm excitation, the best fit was obtained when the contribution of the dimer excitation converges to zero (χ^2 ^= 1.70), indicating that the TRXSS signal measured with 267 nm excitation is also dominated by the contribution of the trimer excitation. Also, when we force the relative fraction of the dimer excitation to be 30%, the fitting quality deteriorates significantly (χ^2 ^= 2.94). In addition, the experimental RDF in *r*-space shown in Figure 6(b) shows the p_1_/p_2_ ratio close to 2, confirming the negligible contribution of the dimer excitation, even with excitation at 267 nm.

As discussed in our previous TRXSS study, the small contribution of the dimer excitation with 267 nm excitation can be attributed to (1) the equilibrium shifted towards the trimer in the 300 mM solution of Au(CN)_2_^−^, (2) the extinction coefficient of trimer much larger than that of dimer at 267 nm, and (3) the X-ray scattering intensity of a single trimer larger than that of a single dimer by at least two times. In our previous TRXSS study,^12^ the population of the excited dimer under our experimental condition was estimated to be only 2.9%. We note that spectroscopic tools are highly sensitive to specific states or chemical species and thus can selectively probe a transient species of even very low concentration as long as its extinction coefficient at the probe wavelengths is sufficiently high. In contrast, TRXSS is sensitive to global molecular structure and its sensitivity increases in proportion to the concentration of a transient species and the masses of its constituent atoms. Therefore, TRXSS signal is not likely to be contaminated by the contributions of minor reaction channels.

## IV. CONCLUSION

In this work, we investigated the reaction mechanism of Au–Au bond formation of [Au(CN)_2_^−^]_3_ trimer induced by 310 nm laser excitation by performing femtosecond TRXSS measurements. We compared the reaction mechanism obtained with excitation at two different wavelengths (310 and 267 nm) and found that the mechanism of Au–Au bond formation in [Au(CN)_2_^−^]_3_ trimer is independent of excitation wavelength. This result validates the assumption used in our previous TRXSS study that the contribution of the dimer excitation to the TRXSS signal measured from the 300 mM solution of Au(CN)_2_^−^ is negligible, regardless of excitation wavelength. Thus, as concluded in our previous TRXSS study, the bent-to-linear transition of [Au(CN)_2_^−^]_3_ trimer occurs within 500 fs.

## Figures and Tables

**TABLE I.** Structural parameters of reaction intermediates used in the data analysis.

Species	R_12_ (Å)	R_23_ (Å)	R_13_ (Å)	R_34_ (Å)
S_0_	3.87 ± 0.04	3.30 ± 0.06	5.56 ± 0.11	···
S_1_ ($T_{1}^{\prime }$)	2.82 ± 0.04	2.81 ± 0.03	5.63 ± 0.09	···
T_1_	2.71 ± 0.03	2.70 ± 0.05	5.41 ± 0.11	···
Tetramer	2.89 ± 0.06	2.62 ± 0.06	···	2.88 ± 0.04

**TABLE II.** Kinetic parameters used in the data analysis.

	S_1_ to T_1_	T_1_ to tetramer	Tetramer to S_0_
Time constant	1.6 ± 0.1 ps	3 ± 0.5 ns	100 ± 20 ns
